# Constructing a linkage–linkage disequilibrium map using dominant-segregating markers

**DOI:** 10.1093/dnares/dsv031

**Published:** 2015-11-29

**Authors:** Xuli Zhu, Leiming Dong, Libo Jiang, Huan Li, Lidan Sun, Hui Zhang, Weiwu Yu, Haokai Liu, Wensheng Dai, Yanru Zeng, Rongling Wu

**Affiliations:** 1 Center for Computational Biology, College of Biological Sciences and Technology, Beijing Forestry University, Beijing 100083, China; 2 The Nurturing Station for the State Key Laboratory of Subtropical Silviculture, Zhejiang A&F University, Lin'an, Zhejiang 311300, China; 3 National Engineering Research Center for Floriculture, Beijing Forestry University, Beijing 10083, China; 4 Center for Statistical Genetics, The Pennsylvania State University, Hershey, PA 17033, USA

**Keywords:** linkage, linkage disequilibrium, linkage–linkage disequilibrium map

## Abstract

The relationship between linkage disequilibrium (LD) and recombination fraction can be used to infer the pattern of genetic variation and evolutionary process in humans and other systems. We described a computational framework to construct a linkage–LD map from commonly used biallelic, single-nucleotide polymorphism (SNP) markers for outcrossing plants by which the decline of LD is visualized with genetic distance. The framework was derived from an open-pollinated (OP) design composed of plants randomly sampled from a natural population and seeds from each sampled plant, enabling simultaneous estimation of the LD in the natural population and recombination fraction due to allelic co-segregation during meiosis. We modified the framework to infer evolutionary pasts of natural populations using those marker types that are segregating in a dominant manner, given their role in creating and maintaining population genetic diversity. A sophisticated two-level EM algorithm was implemented to estimate and retrieve the missing information of segregation characterized by dominant-segregating markers such as single methylation polymorphisms. The model was applied to study the relationship between linkage and LD for a non-model outcrossing species, a gymnosperm species, *Torreya grandis*, naturally distributed in mountains of the southeastern China. The linkage–LD map constructed from various types of molecular markers opens a powerful gateway for studying the history of plant evolution.

## 1. Introduction

Linkage disequilibrium (LD), a concept to describe the non-random association of alleles at different loci, has been a focus of population genetic studies during the last several decades.^1^ However, since LD is affected by many evolutionary forces, the use of LD alone to infer the genetic structure of populations may generate spurious results.^2^ For this reason, how LD can be served as a more efficient tool has been one of the most important issues in population and evolutionary genetics. One of the strategies to resolve this issue is constructing a LD map from which to infer population history by visualizing the decline pattern of LD with genetic distance. This strategy has been widely used in human genetics^3,4,5^ and increasingly recognized in other species.^6,7^ Many of these LD maps are constructed from the relationship of pairwise LD with the physical distance of the same marker pair, which do not estimate the frequency of recombination between marker loci.

The basic principle by which LD is used for historical inference results from its relationship with the recombination rate.^1^ Therefore, the estimation of the linkage, apart from estimating LD, is an essential step towards constructing a LD map. Wu and Zeng^8^ pioneered the application of a sampling design to simultaneously estimate these two parameters. By sampling parents randomly from a natural population and the seeds of the sampled parents, this design constructs a two-level hierarchic structure of molecular data, which enables the characterization of how different markers are associated in the original population and how the markers co-transmit their alleles in a Mendelian fashion from the parent to offspring. Lou et al.^9^ derived a close-form EM algorithm to estimate the LD and recombination rate within a unifying framework. Such a joint linkage–LD analysis has been applied to the genetic mapping of complex traits, leading to the identification of biologically validated quantitative trait loci (QTLs) for drought resistance in maize.^10^ More recently, this strategy has been modified to accommodate to the estimation of genetic imprinting^11^ and genetic variance.^12^ Pikkuhookana and Sillanpaa^13^ implemented a Bayesian algorithm for parameter estimation from this strategy.

In this article, we described a general computational framework built on Wu and Zeng's^8^ open-pollinated design to construct a linkage–LD map using biallelic co-dominant markers. To make this framework more useful for a broader area of applications, we extended it to enable the utilization of dominant-segregating markers. Several recent studies have shown that epigenetic variation provides a source for the generation of phenotypic diversity in natural populations^14,15^ and also epigenetic marks, such as differential cytosine methylation, may be inherited and have experienced the pressure of natural selection.^16^ Thus, it has become increasingly important to construct a more comprehensive linkage–LD map by including methylation markers. In epigenetic population studies, there are many ways to score and analyse methylation-sensitive amplification polymorphisms, of which one common approach is to score those fragments that stay unmethylated as 1 and all others methylated as 0. This scoring approach leads to the segregation pattern of the so-called single methylation polymorphism (SMP) markers equivalent to that of dominant genetic markers.^17^ Lu et al.^18^ found a possibility of using three-point analysis to enhance the precision and power of linkage detection for dominant markers. Likewise, Li et al.^19^ developed a three-point analysis to analyse LD among three dominant markers and establish a procedure for testing and estimating multiple disequilibria at different orders. However, the simultaneous estimates of LD and recombination fraction between dominant markers are methodologically challenging, because their genotypes can little explain the information of allelic segregation. We implemented a two-level EM algorithm for joint linkage and LD analysis by modelling and retrieving the unobservable feature of segregating genotypes for dominant-inherited markers. An example was demonstrated to show the utility and usefulness of the model by analysing a real data collected from an OP design of an outcrossing species, *Torreya grandis*, naturally distributed in mountains of the southeastern China.

## 2. Model

### 2.1. Sampling strategy

From a natural plant population at Hardy–Weinberg equilibrium (HWE), we randomly sample *n* unrelated maternal individuals and open-pollinated seeds from each sampled plant. This constitutes a two-level hierarchic sampling setting in which both parental plants and their offspring are genotyped by the same set of molecular markers. Consider a pair of biallelic markers **A** and **B**, which generate nine joint genotypes, *AABB* (coded as 1), *AABb* (coded as 2), …, *aabb* (coded as 9). Let *n_i_* denotes the number of mother plants with marker genotype *i*, and $n_{i}^{j}$ denotes the number of offspring with marker genotype *j* derived from mother genotype *i*. Depending on the genotype of a mother, all offspring from her have different numbers of marker genotypes. Table 1 tabulates genotypic observations for the two-level hierarchic setting.

**Table 1.** Data structure of two co-dominant markers typed for a panel of half-sib families, each composed of the mother and offspring, sampled at random from a natural population

Grp	Family		Offspring								
	Mother	Num.	*AABB*	*AABb*	*AAbb*	*AaBB*	*AaBb*	*Aabb*	*aaBB*	*aaBb*	*aabb*
1	*AABB*	$n_{1}$	$n_{1}^{1}$	$n_{1}^{2}$		$n_{1}^{4}$	$n_{1}^{5}$				
2	*AABb*	$n_{2}$	$n_{2}^{1}$	$n_{2}^{2}$	$n_{2}^{3}$	$n_{2}^{4}$	$n_{2}^{5}$	$n_{2}^{6}$			
3	*AAbb*	$n_{3}$		$n_{3}^{2}$	$n_{3}^{3}$		$n_{3}^{5}$	$n_{3}^{6}$			
4	*AaBB*	$n_{4}$	$n_{4}^{1}$	$n_{4}^{2}$		$n_{4}^{4}$	$n_{4}^{5}$		$n_{4}^{7}$	$n_{4}^{8}$	
5	*AaBb*	$n_{5}$	$n_{5}^{1}$	$n_{5}^{2}$	$n_{5}^{3}$	$n_{5}^{4}$	$n_{5}^{5}$	$n_{5}^{6}$	$n_{5}^{7}$	$n_{5}^{8}$	$n_{5}^{9}$
6	*Aabb*	$n_{6}$		$n_{6}^{2}$	$n_{6}^{3}$		$n_{6}^{5}$	$n_{6}^{6}$		$n_{6}^{8}$	$n_{6}^{9}$
7	*aaBB*	$n_{7}$				$n_{7}^{4}$	$n_{7}^{5}$		$n_{7}^{7}$	$n_{7}^{8}$	
8	*aaBb*	$n_{8}$				$n_{8}^{4}$	$n_{8}^{5}$	$n_{8}^{6}$	$n_{8}^{7}$	$n_{8}^{8}$	$n_{8}^{9}$
9	*aabb*	$n_{9}$					$n_{9}^{5}$	$n_{9}^{6}$		$n_{9}^{8}$	$n_{9}^{9}$

Let *p_AB_*, *p_Ab_*, *p_aB_* and *p_ab_* denote haplotype frequencies for *AB*, *Ab*, *aB* and *ab*, respectively. The four haplotype frequencies are expressed as

$$\begin{array}{c} \;p_{AB}=\;p_{A}\;p_{B}+D\\ \;p_{Ab}=\;p_{A}\;p_{b}-D\\ \;p_{aB}=\;p_{a}\;p_{B}-D\\ \;p_{ab}=\;p_{a}\;p_{b}+D \end{array}$$

where allele frequencies are defined as *p_A_* and *p_a_* (*p_A_* + *p_a_* = 1) for marker **A** and *p_B_* and *p_b_* (*p_B_* + *p_b_* = 1) for marker **B**, respectively, and *D* is the LD between the two markers. Under the assumption of HWE, the expected frequency of two-marker genotype *i* in the parental population (*P_i_*) is expressed as the product of the two corresponding haplotype frequencies. Based on the principle of co-transmission of two genes from a parent to its progeny, we derived the expected frequency of two-marker genotype *j* in the progeny population from mother genotype *i* ($P_{i}^{j}$), expressed as a function of the recombination fraction *θ* for the double heterozygous mother genotype. All these maternal and offspring genotype frequencies are given in Table 2.

**Table 2.** Mating frequencies of mother and offspring genotype frequencies per family for two co-dominant markers sampled from a natural population

No.			Offspring									
	Maternal mating		*AABB*	*AABb*	*AAbb*	*AaBB*	*AaBb*		*Aabb*	*aaBB*	*aaBb*	*aabb*
	Genotype	Frequency	*AB|AB*	*AB|Ab*	*Ab|Ab*	*AB|aB*	*AB|ab*	*Ab|aB*	*Ab|ab*	*aB|aB*	*aB|ab*	*ab|ab*
1	*AABB*	$p_{AB}^{2}$	$\;p_{AB}$	$\;p_{Ab}$		$\;p_{aB}$	$\;p_{ab}$					
2	*AABb*	$2\;p_{AB}\;p_{Ab}$	$\frac{1}{2}\;p_{AB}$	$\frac{1}{2}\;p_{AB}+\frac{1}{2}\;p_{Ab}$	$\frac{1}{2}\;p_{Ab}$	$\frac{1}{2}\;p_{aB}$	$\frac{1}{2}\;p_{ab}+\frac{1}{2}\;p_{aB}$		$\frac{1}{2}\;p_{ab}$			
3	*AAbb*	$p_{Ab}^{2}$		$\;p_{AB}$	$\;p_{Ab}$		$\;p_{aB}$		$\;p_{ab}$			
4	*AaBB*	$2\;p_{AB}\;p_{aB}$	$\frac{1}{2}\;p_{AB}$	$\frac{1}{2}\;p_{Ab}$		$\frac{1}{2}\;p_{AB}+\frac{1}{2}\;p_{aB}$	$\frac{1}{2}\;p_{ab}+\frac{1}{2}\;p_{Ab}$			$\frac{1}{2}\;p_{aB}$	$\frac{1}{2}\;p_{ab}$	
5	*AaBb*	$\left\{\begin{array}{c} 2\;p_{AB}\;p_{ab}\;\\ +\;\\ 2\;p_{Ab}\;p_{aB}\; \end{array}\right.$	$\omega_{1}\;p_{AB}$	$\begin{array}{ll} & \omega_{1}\;p_{Ab}\\ & +\\ & \omega_{2}\;p_{AB} \end{array}$	$\omega_{2}\;p_{Ab}$	$\begin{array}{ll} & \omega_{1}\;p_{aB}\\ & +\\ & \omega_{2}\;p_{AB} \end{array}$	$\begin{array}{ll} & \omega_{1}(\;p_{ab}+\;p_{AB})\\ & +\\ & \omega_{2}(\;p_{aB}+\;p_{Ab}) \end{array}$		$\begin{array}{ll} & \omega_{1}\;p_{Ab}\\ & +\\ & \omega_{2}\;p_{ab} \end{array}$	$\omega_{2}\;p_{aB}$	$\begin{array}{ll} & \omega_{1}\;p_{aB}\\ & +\\ & \omega_{2}\;p_{ab} \end{array}$	$\omega_{1}\;p_{ab}$
6	*Aabb*	$2\;p_{Ab}\;p_{ab}$		$\frac{1}{2}\;p_{AB}$	$\frac{1}{2}\;p_{Ab}$		$\frac{1}{2}\;p_{AB}+\frac{1}{2}\;p_{aB}$		$\frac{1}{2}\;p_{Ab}+\frac{1}{2}\;p_{ab}$		$\frac{1}{2}\;p_{aB}$	$\frac{1}{2}\;p_{ab}$
7	*aaBB*	$p_{aB}^{2}$				$\;p_{AB}$	$\;p_{Ab}$			$\;p_{aB}$	$\;p_{ab}$	
8	*aaBb*	$2\;p_{aB}\;p_{ab}$				$\frac{1}{2}\;p_{AB}$	$\frac{1}{2}\;p_{AB}+\frac{1}{2}\;p_{Ab}$		$\frac{1}{2}\;p_{Ab}$	$\frac{1}{2}\;p_{aB}$	$\frac{1}{2}\;p_{ab}+\frac{1}{2}\;p_{aB}$	$\frac{1}{2}\;p_{ab}$
9	*aabb*	$p_{ab}^{2}$					$\;p_{AB}$		$\;p_{Ab}$		$\;p_{aB}$	$\;p_{ab}$

Maternal genotype *AaBb* (no. 5) contains a mix of different diplotypes that are encompassed by a box.

### 2.2. The co-dominant model

#### 2.2.1. Likelihood

We use **M***_m_* and **M***_o_* to denote observed maternal genotypes and offspring genotypes for markers **A** and **B**. Let (**Ω***_p_*, *θ*) denote the unknown parameters including all haplotype frequencies (arrayed in **Ω***_p_*) and the recombination fraction *θ*. A unifying log-likelihood that integrates two-level maternal and progeny genotype data can be expressed as

(1) $$L((\Omega_{p},\theta )|(M_{m},M_{o}))=\text{constant}+\begin{array}{c} \text{Upper level}\;\text{Lower level}\;\\ n_{i}\text{log(}P_{i})\;+\;n_{i}^{j}\text{log}(P_{i}^{j})\;\\ \text{Maternal}\;\text{Offspring}\; \end{array}$$

where the first term of the right side is the upper level likelihood constructed by maternal genotype observations and haplotype frequencies **Ω***_p_* that form expected maternal genotype frequencies (Table 2) and the second term is the lower level likelihood constructed by maternal and offspring genotypes, haplotype frequencies **Ω***_p_* and the recombination fraction *θ*. The upper level likelihood is constructed by mother genotype observations and expected mother genotype frequencies, expressed as

(2) $$\begin{array}{ll} \log\;L(\Omega_{p}|M_{m})= & \;\text{constant}+n_{1}\log(\;p_{AB}^{2})+n_{2}\log(2\;p_{AB}\;p_{Ab})\\ & +n_{3}\log(\;p_{Ab}^{2})+n_{4}\log(2\;p_{AB}\;p_{aB})\\ & +n_{5}\log(2\;p_{AB}\;p_{ab}+2\;p_{Ab}\;p_{aB})+n_{6}\log(2\;p_{Ab}\;p_{ab})\\ & +n_{7}\log(\;p_{aB}^{2})+n_{8}\log(2\;p_{aB}\;p_{ab})+n_{9}\log(\;p_{ab}^{2}) \end{array}$$

For double heterozygote *AaBb*, its observed genotype may be derived from two possible diplotypes, *AB*|*ab* (with probability of *p_AB_p_ab_*) or *Ab*|*aB* (with probability of *p_Ab_p_aB_*), where the vertical lines are used to separate the two underlying haplotypes of a diplotype. For a given parental genotype combination, a certain group of offspring genotypes is produced. For a mother with genotype *AaBb*, there will be two possible diplotypes, *AB|ab* and *Ab|aB*, whose relative frequencies are

(3) $$\phi =\frac{\;p_{AB}\;p_{ab}}{\;p_{AB}\;p_{ab}+\;p_{Ab}\;p_{aB}},\;1-\phi =\frac{\;p_{Ab}\;p_{aB}}{\;p_{AB}\;p_{ab}+\;p_{Ab}\;p_{aB}},$$

respectively. Both the diplotypes will produce haplotypes *AB*, *Ab*, *aB* and *ab* with frequencies defined as follows:

**Table:** 

Parent	*AaBb*	Haplotype			
Diplotype	Frequency	*AB*	*Ab*	*aB*	*ab*
*AB|ab*	*ϕ*	$\frac{1}{2}(1-\theta )$	$\frac{1}{2}\theta$	$\frac{1}{2}\theta$	$\frac{1}{2}(1-\theta )$
*Ab|aB*	1−*ϕ*	$\frac{1}{2}\theta$	$\frac{1}{2}(1-\theta )$	$\frac{1}{2}(1-\theta )$	$\frac{1}{2}\theta$

Let $\omega_{1}=\frac{1}{2}\phi (1-\theta )+\frac{1}{2}(1-\phi )\theta ,\omega_{2}=\frac{1}{2}\phi \theta +\frac{1}{2}(1-\phi )(1-\theta )$. Thus, overall haplotype frequencies produced by this parent are calculated as $\omega_{1}$for *AB* or *ab* and $\omega_{2}$ for *Ab* or *aB*.

Based on the information about genetic segregation in each family, the lower lever likelihood is constructed as

(4) $$\begin{array}{ll} & \log L(\Omega_{g}|M_{m},M_{o},\Omega_{p})=\text{constant}\\ & \;+n_{1}^{1}\log(\;p_{AB})+n_{1}^{2}\log(\;p_{Ab})+n_{1}^{4}\log(\;p_{aB})+n_{1}^{5}\log(\;p_{ab})\\ & \;+n_{2}^{1}\log(\frac{1}{2}\;p_{AB})+n_{2}^{2}\log(\frac{1}{2}\;p_{AB}+\frac{1}{2}\;p_{Ab})+n_{2}^{3}\log(\frac{1}{2}\;p_{Ab})\\ & \;+n_{2}^{4}\log(\frac{1}{2}\;p_{aB})+n_{2}^{5}\log(\frac{1}{2}\;p_{ab}+\frac{1}{2}\;p_{aB})+n_{2}^{6}\log(\frac{1}{2}\;p_{ab})\\ & \;+n_{3}^{2}\log(\;p_{AB})+n_{3}^{3}\log(\;p_{Ab})+n_{3}^{5}\log(\;p_{aB})+n_{3}^{6}\log(\;p_{ab})\\ & \;+n_{4}^{1}\log(\frac{1}{2}\;p_{AB})+n_{4}^{2}\log(\frac{1}{2}\;p_{Ab})+n_{4}^{4}\log(\frac{1}{2}\;p_{AB}+\frac{1}{2}\;p_{aB})\\ & \;+n_{4}^{5}\log(\frac{1}{2}\;p_{ab}+\frac{1}{2}\;p_{Ab})+n_{4}^{7}\log(\frac{1}{2}p_{aB}^{\;})+n_{4}^{8}\log(\frac{1}{2}p_{ab}^{\;})\\ & \;+n_{5}^{1}\log(\omega_{1}\;p_{AB})+n_{5}^{2}\log(\omega_{1}\;p_{Ab}+\omega_{2}\;p_{AB})+n_{5}^{3}\log(\omega_{2}\;p_{Ab})\\ & \;+n_{5}^{4}\log(\omega_{1}\;p_{aB}+\omega_{2}\;p_{AB})+n_{5}^{5}\log(\omega_{1}(\;p_{ab}+\;p_{AB})+\omega_{2}(\;p_{aB}+\;p_{Ab}))\\ & \;+n_{5}^{6}\log(\omega_{1}\;p_{Ab}+\omega_{2}\;p_{ab})+n_{5}^{7}\log(\omega_{2}\;p_{aB})+n_{5}^{8}\log(\omega_{1}\;p_{aB}+\omega_{2}\;p_{ab})\\ & \;+n_{5}^{9}\log(\omega_{1}\;p_{ab})+n_{6}^{2}\log(\frac{1}{2}\;p_{AB})+n_{6}^{3}\log(\frac{1}{2}\;p_{Ab})+n_{6}^{5}\log(\frac{1}{2}\;p_{AB}+\frac{1}{2}\;p_{aB})\\ & \;+n_{6}^{6}\log(\frac{1}{2}\;p_{Ab}+\frac{1}{2}\;p_{ab})+n_{6}^{8}\log(\frac{1}{2}\;p_{aB})+n_{6}^{9}\log(\frac{1}{2}\;p_{ab})\\ & \;+n_{7}^{4}\log(\;p_{AB})+n_{7}^{5}\log(\;p_{Ab})+n_{7}^{7}\log(\;p_{aB})+n_{7}^{8}\log(\;p_{ab})\\ & \;+n_{8}^{4}\log(\frac{1}{2}\;p_{AB})+n_{8}^{5}\log(\frac{1}{2}\;p_{AB}+\frac{1}{2}\;p_{Ab})+n_{8}^{6}\log(\frac{1}{2}\;p_{Ab})\\ & \;+n_{8}^{7}\log(\frac{1}{2}\;p_{aB})+n_{8}^{8}\log(\frac{1}{2}\;p_{ab}+\frac{1}{2}\;p_{aB})+n_{8}^{9}\log(\frac{1}{2}\;p_{ab})\\ & \;+n_{9}^{6}\log(\;p_{AB})+n_{9}^{7}\log(\;p_{Ab})+n_{9}^{8}\log(\;p_{aB})+n_{9}^{9}\log(\;p_{ab}) \end{array}$$

Below, an algorithmic procedure will be described to estimate the parameters that define the likelihood.

#### 2.2.2. Estimation

We implement two EM algorithms to estimate the unknown parameters. The first is implemented to estimate the haplotype frequencies and therefore allelic frequencies and linkage disequilibria by jointly maximizing log-likelihoods (2) and (4). The second is implemented to estimate the recombination fraction that is contained with double heterozygote by maximizing the log-likelihood (4). In the E step of the second EM algorithm, we calculate the probability with which a considered haplotype produced by double heterozygote parent is the recombinant type using

(5) $$\begin{array}{ccc} \psi_{1}=(1-\phi )\theta \; & \psi_{3}=(1-\phi )\theta +\phi (1-\theta )\; & \;\text{for haplotype }AB\;\text{or }a\text{b}\;\\ \psi_{2}=\phi \theta \; & \psi_{4}=\phi \theta +(1-\phi )(1-\theta )\; & \;\text{for haplotype }Ab\;\text{or }a\text{B}\; \end{array}$$

In the M step, the estimate of the recombination fraction is obtained by

(6) $$\theta =\frac{m}{M}$$

where *m* equals the sum of following terms,

$$\begin{array}{ll} & \frac{\psi_{1}}{\psi_{3}}(n_{5}^{1}+n_{5}^{9})+\frac{\psi_{2}}{\psi_{4}}(n_{5}^{3}+n_{5}^{7})\\ & \;+\left(\frac{\psi_{1}\;p_{Ab}}{\psi_{3}\;p_{Ab}+\psi_{4}\;p_{AB}}+\frac{\psi_{2}\;p_{AB}}{\psi_{3}\;p_{Ab}+\psi_{4}\;p_{AB}}\right)n_{5}^{2}\\ & \;+\left(\frac{\psi_{1}\;p_{aB}}{\psi_{3}\;p_{aB}+\psi_{4}\;p_{AB}}+\frac{\psi_{2}\;p_{AB}}{\psi_{3}\;p_{aB}+\psi_{4}\;p_{AB}}\right)n_{5}^{4}\\ & \;+\left(\frac{\psi_{1}\;p_{Ab}}{\psi_{3}\;p_{Ab}+\psi_{4}\;p_{ab}}+\frac{\psi_{2}\;p_{ab}}{\psi_{3}\;p_{Ab}+\psi_{4}\;p_{ab}}\right)n_{5}^{6}\\ & \;+\left(\frac{\psi_{1}\;p_{aB}}{\psi_{3}\;p_{aB}+\psi_{4}\;p_{ab}}+\frac{\psi_{2}\;p_{ab}}{\psi_{3}\;p_{aB}+\psi_{4}\;p_{ab}}\right)n_{5}^{8}\\ & \;+\left(\begin{array}{ll} & \frac{\psi_{1}(\;p_{AB}+\;p_{ab})}{\psi_{3}(\;p_{AB}+\;p_{ab})+\psi_{4}(\;p_{Ab}+\;p_{aB})}\\ & \;+\frac{\psi_{2}(\;p_{Ab}+\;p_{aB})}{\psi_{3}(\;p_{AB}+\;p_{ab})+\psi_{4}(\;p_{Ab}+\;p_{aB})} \end{array}\right)n_{5}^{5} \end{array}$$

and

$$M=n_{5}^{1}+n_{5}^{2}+n_{5}^{3}+n_{5}^{4}+n_{5}^{5}+n_{5}^{6}+n_{5}^{7}+n_{5}^{8}+n_{5}^{9}$$

The E and M steps are iterated between Equations (5) and (6) until convergence.

#### 2.2.3. Hypothesis testing

After genetic parameters are estimated, we test whether the two markers are associated and/or linked on the same genomic region. This can use the following hypotheses:

$$\begin{array}{ll} & H_{0}:D=0\;\text{and }\theta =0.5\\ & H_{1}:\text{At least one of the equalities above does not hold} \end{array}$$

The likelihoods under the *H*_0_ and *H*_1_ are calculated from which a log-likelihood ratio is calculated. By comparing this test statistic with a *χ*^2^ threshold with two degrees of freedom, we can accept or reject *H*_0_.

It is also needed to test the significance of *D* and *θ* separately, showing how the two markers are related. Under the null hypothesis $H_{0}:D=0,$ parental diplotype and genotype frequencies can be simply expressed as a function of allele frequencies which can be estimated with no need of the EM algorithm. Similarly, under the null hypothesis $H_{0}:\theta =0.5$, offspring diplotype and genotype frequencies within each family are simply expressed as function of the Mendelian segregation ratio so that no parameter needs to be estimated.

### 2.3. The dominant model

#### 2.3.1. Estimation

Methylation-sensitive amplification polymorphisms can be scored as a dominant marker.^17^ For a dominant marker, the homozygote *AA* for the dominant allele cannot be distinguished from the heterozygote *Aa*. Thus, these two genotypes are observed as a single ‘phenotype’ (*A_*). For two dominant markers **A** and **B**, some cells for the observations and expected genotype frequencies in Tables 1 and 2 are collapsed in a way as shown in Tables 3 and 4, respectively. Let $n_{\;j_{A}/\;j_{B}}$ denote the observed number of observations of a two-dominant marker genotype *j_A_/j_B_*, *j_A_*= *A_* (coded as 1) or *aa* (coded as 0) and *j_B_*= *B_* (coded as 1) or *bb* (coded as 0), in the parental population. Similarly, let $n_{\;j_{A}/\;j_{B}}^{k_{A}/k_{B}}$ denote the observation of progeny marker genotype *k_A_*/*k_B_* given its parent genotype *j_A_*/*j_B_* (Table 3). Frequencies of offspring genotypes from different mother genotypes are shown in Table 4.

**Table 3.** Data structure of two dominant markers typed for a panel of half-sib families, each composed of the mother and offspring, sampled at random from a natural population

Grp	Family		Offspring			
	Mother	Num.	*A_B_*	*A_bb*	*aaB_*	*aabb*
1	*A_B_*	$n_{1/1}$	$n_{1/1}^{1/1}$	$n_{1/1}^{1/0}$	$n_{1/1}^{0/1}$	$n_{1/1}^{0/0}$
2	*A_bb*	$n_{1/0}$	$n_{1/0}^{1/1}$	$n_{1/0}^{1/0}$	$n_{1/0}^{0/1}$	$n_{1/0}^{0/0}$
3	*aaB_*	$n_{0/1}$	$n_{0/1}^{1/1}$	$n_{0/1}^{1/0}$	$n_{0/1}^{0/1}$	$n_{0/1}^{0/0}$
4	*aabb*	$n_{0/0}$	$n_{0/0}^{1/1}$	$n_{0/0}^{1/0}$	$n_{0/0}^{0/1}$	$n_{0/0}^{0/0}$

*A_ = AA + Aa and B_ = BB + Bb*.

**Table 4.** Mating frequencies of mother and offspring genotype frequencies per family for two dominant markers sampled from a natural population

No.	Parental mating		Offspring			
	Mother	Frequency	*A_B*	*A_bb*	*aaB*	*aabb*
1	*A_B_*	$\left\{\begin{array}{c} \;p_{AB}^{2}\;\\ +\;\\ 2\;p_{AB}\;p_{Ab}\;\\ +\;\\ 2\;p_{AB}\;p_{aB}\;\\ +\;\\ 2\;p_{AB}\;p_{ab}\;\\ +\;\\ 2\;p_{Ab}\;p_{aB}\; \end{array}\right.$	$\left\{\begin{array}{c} \;p_{AB}^{2}\;\\ +\;\\ \;p_{AB}\;p_{Ab}(2\;p_{AB}+\;p_{Ab}+2\;p_{aB}+\;p_{ab})\;\\ +\;\\ \;p_{AB}\;p_{aB}(\;p_{AB}+2\;p_{Ab}+2\;p_{aB}+\;p_{ab})\;\\ +\;\\ 2\omega_{1}\;p_{AB}\;p_{ab}(2\;p_{AB}+\;p_{Ab}+\;p_{aB}+\;p_{ab})\;\\ +\;\\ 2\omega_{2}\;p_{Ab}\;p_{aB}(2\;p_{AB}+\;p_{Ab}+\;p_{aB})\; \end{array}\right.$	$\left\{\begin{array}{c} 0\;\\ +\;\\ \;p_{AB}\;p_{Ab}(\;p_{Ab}+\;p_{ab})\;\\ +\;\\ 0\;\\ +\;\\ 2\omega_{1}\;p_{AB}\;p_{ab}(\;p_{Ab})\;\\ +\;\\ 2\omega_{2}\;p_{Ab}\;p_{aB}(2\;p_{Ab}+\;p_{ab})\; \end{array}\right.$	$\left\{\begin{array}{c} 0\;\\ +\;\\ 0\;\\ +\;\\ \;p_{AB}\;p_{aB}(\;p_{AB}+\;p_{ab})\;\\ +\;\\ 2\omega_{1}\;p_{AB}\;p_{ab}(\;p_{aB})\;\\ +\;\\ 2\omega_{2}\;p_{Ab}\;p_{aB}(\;p_{aB}+\;p_{ab})\; \end{array}\right.$	$\left\{\begin{array}{c} 0\;\\ +\;\\ 0\;\\ +\;\\ 0\;\\ +\;\\ 2\omega_{1}\;p_{AB}\;p_{ab}(\;p_{ab})\;\\ +\;\\ 0\; \end{array}\right.$
2	*A_bb*	$\left\{\begin{array}{c} \;p_{Ab}^{2}\;\\ +\;\\ 2\;p_{Ab}\;p_{ab}\; \end{array}\right.$	$\left\{\begin{array}{c} \;p_{Ab}^{2}(\;p_{AB}+\;p_{aB})\;\\ +\;\\ \;p_{Ab}\;p_{ab}(2\;p_{AB}+\;p_{aB})\; \end{array}\right.$	$\left\{\begin{array}{c} \;p_{Ab}^{2}(\;p_{Ab}+\;p_{ab})\;\\ +\;\\ \;p_{Ab}\;p_{ab}(2\;p_{Ab}+\;p_{ab})\; \end{array}\right.$	$\left\{\begin{array}{c} 0\;\\ +\;\\ p_{Ab}^{\;}p_{ab}^{\;}(\;p_{aB}^{\;})\; \end{array}\right.$	$\left\{\begin{array}{c} 0\;\\ +\;\\ \;p_{Ab}\;p_{ab}(\;p_{ab})\; \end{array}\right.$
3	*aaB_*	$\left\{\begin{array}{c} \;p_{aB}^{2}\;\\ +\;\\ 2\;p_{aB}\;p_{ab}\; \end{array}\right.$	$\left\{\begin{array}{c} \;p_{aB}^{2}(\;p_{AB}+\;p_{Ab})\;\\ +\;\\ \;p_{aB}\;p_{ab}(2\;p_{AB}+\;p_{Ab})\; \end{array}\right.$	$\left\{\begin{array}{c} 0\;\\ +\;\\ \;p_{aB}\;p_{ab}(\;p_{Ab})\; \end{array}\right.$	$\left\{\begin{array}{c} (\;p_{aB}+\;p_{ab})\;\\ +\;\\ \;p_{aB}\;p_{ab}(2\;p_{aB}+\;p_{ab})\; \end{array}\right.$	$\left\{\begin{array}{c} 0\;\\ +\;\\ \;p_{aB}\;p_{ab}(\;p_{ab})\; \end{array}\right.$
4	*aabb*	$p_{ab}^{2}$	$\;p_{AB}$	$\;p_{Ab}$	$\;p_{aB}$	$\;p_{ab}$

$\omega_{1}=\frac{1}{2}\phi (1-\theta )+\frac{1}{2}(1-\phi )\theta ,\omega_{2}=\frac{1}{2}\phi \theta +\frac{1}{2}(1-\phi )(1-\theta ),\phi =\;p_{AB}\;p_{ab}/(\;p_{AB}\;p_{ab}+\;p_{Ab}\;p_{aB})$.

A two-level hierarchical likelihood (1) is formulated to jointly estimate haplotype frequencies and recombination fraction by implementing two EM algorithms. The first EM algorithm is used to estimate haplotype frequencies by jointly maximizing the upper and lower level likelihood, whereas the second EM algorithm used to estimate the recombination by maximizing the lower level likelihood. Here, we show how the second EM algorithm is implemented to estimate the recombination fraction. In the E step, we calculate the overall frequencies of the genotype with the progeny cells (Table 4) using

(7) $$\begin{array}{ll} \Phi_{1} & =\frac{2\omega_{1}\;p_{AB}(\;p_{AB}\;p_{ab}+\;p_{Ab}\;p_{aB})}{\delta_{1}}\\ \Phi_{2} & =\frac{2(\omega_{1}\;p_{Ab}+\omega_{2}\;p_{AB})(\;p_{AB}\;p_{ab}+\;p_{Ab}\;p_{aB})}{\delta_{1}}\\ \Phi_{3} & =\frac{2\omega_{2}\;p_{Ab}(\;p_{AB}\;p_{ab}+\;p_{Ab}\;p_{aB})}{\delta_{2}}\\ \Phi_{4} & =\frac{2(\omega_{1}\;p_{aB}+\omega_{2}\;p_{AB})(\;p_{AB}\;p_{ab}+\;p_{Ab}\;p_{aB})}{\delta_{1}}\\ \Phi_{5} & =\frac{2(\omega_{1}\;p_{ab}+\omega_{1}\;p_{AB}+\omega_{2}\;p_{aB}+\omega_{2}\;p_{Ab})(\;p_{AB}\;p_{ab}+\;p_{Ab}\;p_{aB})}{\delta_{1}}\\ \Phi_{6} & =\frac{2(\omega_{1}\;p_{Ab}+\omega_{2}\;p_{ab})(\;p_{AB}\;p_{ab}+\;p_{Ab}\;p_{aB})}{\delta_{2}}\\ \Phi_{7} & =\frac{2\omega_{2}\;p_{aB}(\;p_{AB}\;p_{ab}+\;p_{Ab}\;p_{aB})}{\delta_{3}}\\ \Phi_{8} & =\frac{2(\omega_{1}\;p_{aB}+\omega_{2}\;p_{ab})(\;p_{AB}\;p_{ab}+\;p_{Ab}\;p_{aB})}{\delta_{3}} \end{array}$$

where

$$\begin{array}{ll} \delta_{1}= & \;\;p_{AB}^{2}+\;p_{AB}\;p_{Ab}(2\;p_{AB}+\;p_{Ab}+2\;p_{aB}+\;p_{ab})\\ & +\;p_{AB}\;p_{aB}(\;p_{AB}+2\;p_{Ab}+2\;p_{aB}+\;p_{ab})\\ & \;+2\omega_{1}\;p_{AB}\;p_{ab}(2\;p_{AB}+\;p_{Ab}+\;p_{aB}+\;p_{ab})\\ & +2\omega_{2}\;p_{Ab}\;p_{aB}(2\;p_{AB}+\;p_{Ab}+\;p_{aB})\\ \delta_{2}= & \;\;p_{AB}\;p_{Ab}(\;p_{Ab}+\;p_{ab})\\ & +2\omega_{1}\;p_{AB}\;p_{ab}(\;p_{Ab})+2\omega_{2}\;p_{Ab}\;p_{aB}(2\;p_{Ab}+\;p_{ab})\\ \delta_{3}= & \;\;p_{AB}\;p_{aB}(\;p_{AB}+\;p_{ab})+2\omega_{1}\;p_{AB}\;p_{ab}(\;p_{aB})\\ & +2\omega_{2}\;p_{Ab}\;p_{aB}(\;p_{aB}+\;p_{ab}) \end{array}$$

$$\begin{array}{ll} & \omega_{1}=\frac{1}{2}\phi (1-\theta )+\frac{1}{2}(1-\phi )\theta \\ & \omega_{2}=\frac{1}{2}\phi \theta +\frac{1}{2}(1-\phi )(1-\theta ); \end{array}$$

We interpret $\Phi_{1}$ as a probability that the offspring genotype is *AABB* and the mother genotype is *AaBb* while both offspring and mother genotypes are observed as *A_B_*. The other Φ's can be interpreted in a similar way.

In the M step, we estimate the recombination fraction by

(8) $$\theta =\frac{m}{M}$$

where $M=n_{1/1}^{1/1}(\Phi_{1}+\Phi_{2}+\Phi_{4}+\Phi_{5})+n_{1/1}^{1/0}(\Phi_{3}+\Phi_{6})+n_{1/1}^{0/1}(\Phi_{7}\;+\Phi_{8})+n_{1/1}^{0/0}$ and *m* is the sum of following terms, expressed as

$$\begin{array}{ll} & \;\frac{\psi_{1}}{\psi_{3}}(n_{1/1}^{1/1}\Phi_{1}+n_{1/1}^{0/0})+\frac{\psi_{2}}{\psi_{4}}(n_{1/1}^{1/0}\Phi_{3}+n_{1/1}^{0/1}\Phi_{7})\\ & \;+\left(\frac{\psi_{1}\;p_{Ab}}{\psi_{3}\;p_{Ab}+\psi_{4}\;p_{AB}}+\frac{\psi_{2}\;p_{AB}}{\psi_{3}\;p_{Ab}+\psi_{4}\;p_{AB}}\right)n_{1/1}^{1/1}\Phi_{2} \end{array}$$

$$\;\begin{array}{ll} & +\left(\frac{\psi_{1}\;p_{aB}}{\psi_{3}\;p_{aB}+\psi_{4}\;p_{AB}}+\frac{\psi_{2}\;p_{AB}}{\psi_{3}\;p_{aB}+\psi_{4}\;p_{AB}}\right)n_{1/1}^{1/1}\Phi_{4}\\ & +\left(\frac{\psi_{1}\;p_{Ab}}{\psi_{3}\;p_{Ab}+\psi_{4}\;p_{ab}}+\frac{\psi_{2}\;p_{ab}}{\psi_{3}\;p_{Ab}+\psi_{4}\;p_{ab}}\right)n_{1/1}^{1/0}\Phi_{6} \end{array}$$

$$\begin{array}{ll} & +\left(\frac{\psi_{1}\;p_{aB}}{\psi_{3}\;p_{aB}+\psi_{4}\;p_{ab}}+\frac{\psi_{2}\;p_{ab}}{\psi_{3}\;p_{aB}+\psi_{4}\;p_{ab}}\right)n_{1/1}^{0/1}\Phi_{8}\\ & +(\begin{array}{l} \frac{\psi_{1}(\;p_{AB}+\;p_{ab})}{\psi_{3}(\;p_{AB}+\;p_{ab})+\psi_{4}(\;p_{Ab}+\;p_{aB})}+\frac{\psi_{2}(\;p_{Ab}+\;p_{aB})}{\psi_{3}(\;p_{AB}+\;p_{ab})+\psi_{4}(\;p_{Ab}+\;p_{aB})})n_{1/1}^{1/1}\Phi_{5} \end{array} \end{array}$$

with *ψ*_1_ and *ψ*_2_ defined as the probabilities with which a considered haplotype produced by a double heterozygote parent *AaBb* is the recombinant type, i.e.

$$\begin{array}{cc} \psi_{1}=(1-\phi )\theta \; & \;\text{for haplotype }AB\text{or }a\text{b}\;\\ \psi_{2}=\phi \theta \; & \;\text{for haplotype }Ab\text{or }a\text{B}\; \end{array}$$

and with *ψ*_3_ and *ψ*_4_ defined as the probabilities with which the double heterozygote parent *AaBb* produce haplotype *AB, ab* or *Ab, aB*, i.e.

$$\begin{array}{cc} \psi_{3}=(1-\phi )\theta +\phi (1-\theta )\; & \;\text{for haplotype }AB\text{or }a\text{b}\;\\ \psi_{4}=\phi \theta +(1-\phi )(1-\theta )\; & \;\text{for haplotype }Ab\text{or }a\text{B}\; \end{array}$$

where *ϕ* is defined in Equation (3).

#### 2.3.2. Hypothesis testing

We formulate the hypothesis tests for the significance of the LD and linkage. The estimation of allele frequencies of two dominant markers under the null hypothesis of no LD should also be based on the EM algorithm; i.e. in the E step, we calculate

(9) $$\begin{array}{cc} \Phi_{A}=\frac{2}{2-\;p_{A}},\; & \Phi_{a}=\frac{2\;p_{a}}{1+pa},\;\\ \Phi_{B}=\frac{2}{2-\;p_{B}},\; & \Phi_{b}=\frac{2\;p_{b}}{1+\;p_{b}},\; \end{array}$$

In the M step, we estimate the allele frequencies of markers **A** and **B** by using

(10) $$\begin{array}{ll} & \;p_{A}=\frac{\Phi_{A}(n_{1/1}+n_{1/0})}{2n},\;\;p_{a}=\frac{2(n_{0/1}+n_{0/0})+\Phi_{a}(n_{1/1}+n_{1/0})}{2n},\\ & \;p_{B}=\frac{\Phi_{B}(n_{1/1}+n_{0/1})}{2n},\;\;p_{b}=\frac{2(n_{1/0}+n_{0/0})+\Phi_{b}(n_{1/1}+n_{0/1})}{2n}. \end{array}$$

The E and M steps between Equations (9) and (10) are iterated until the estimates are stable. The log-likelihood ratio under the null and alternative hypotheses is calculated and compared with a threshold determined from a *χ*^2^ distribution.

## 3. Application

We used a real data analysis to demonstrate how the model is used to construct a linkage–LD map. According to Wu and Zeng's^8^ design, we sampled a natural population of *Torreya grandis* distributed in the southeastern China.^20^ In spite of the economic value of *T. grandis*, this species has not been extensively studied in population genetics. Zeng et al.^21^ constructed a first low-density genetic map for genus *Torreya* using an open-pollinated progeny derived from half-sib seeds of a landrace *T. grandis* ‘*Merrillii*’, providing basic information for marker genotyping of this species. We sampled 50 unrelated trees randomly from a natural population of *T. grandis* and 20 progeny for each sampled tree. In total, we obtained (50 + 50 × 20) = 1,050 trees, which were genotyped by 233 sequence-related amplified polymorphism (SRAP) markers. SRAPs are dominant-segregating markers,^22^ providing an excellent demonstration for the practical utility of our model. This data set constitutes a two-level hierarchic framework with a high level from the parents and a lower level from the progeny. We analysed each pair of these markers using the dominant model to simultaneously estimate the LD and recombination fraction and further test the significance of these two parameters.

Of 233 × 232/2 = 27,028 pairs, 5,733 (21.21%) display significant non-random associations, and 2,140 (7.92%) are significantly linked. It was seen that much fewer pairs (1,239 or 4.58%) are both associated in the original natural population and linked when they co-transmitted during meiosis from the parent to progeny. All this suggests that, for many marker pairs, significant associations are inconsistent with significant linkage. In other words, a pair of unlinked markers may be associated with each other, and also a pair of linked markers may not necessarily have a significant LD. Significant associations of unlinked markers may be due to the impact of some recent evolutionary forces on these markers, whereas the absence of associations between linked markers implies that this particular region of the genome has experienced the random mating of numerous generations.^23^

**Figure 1.**
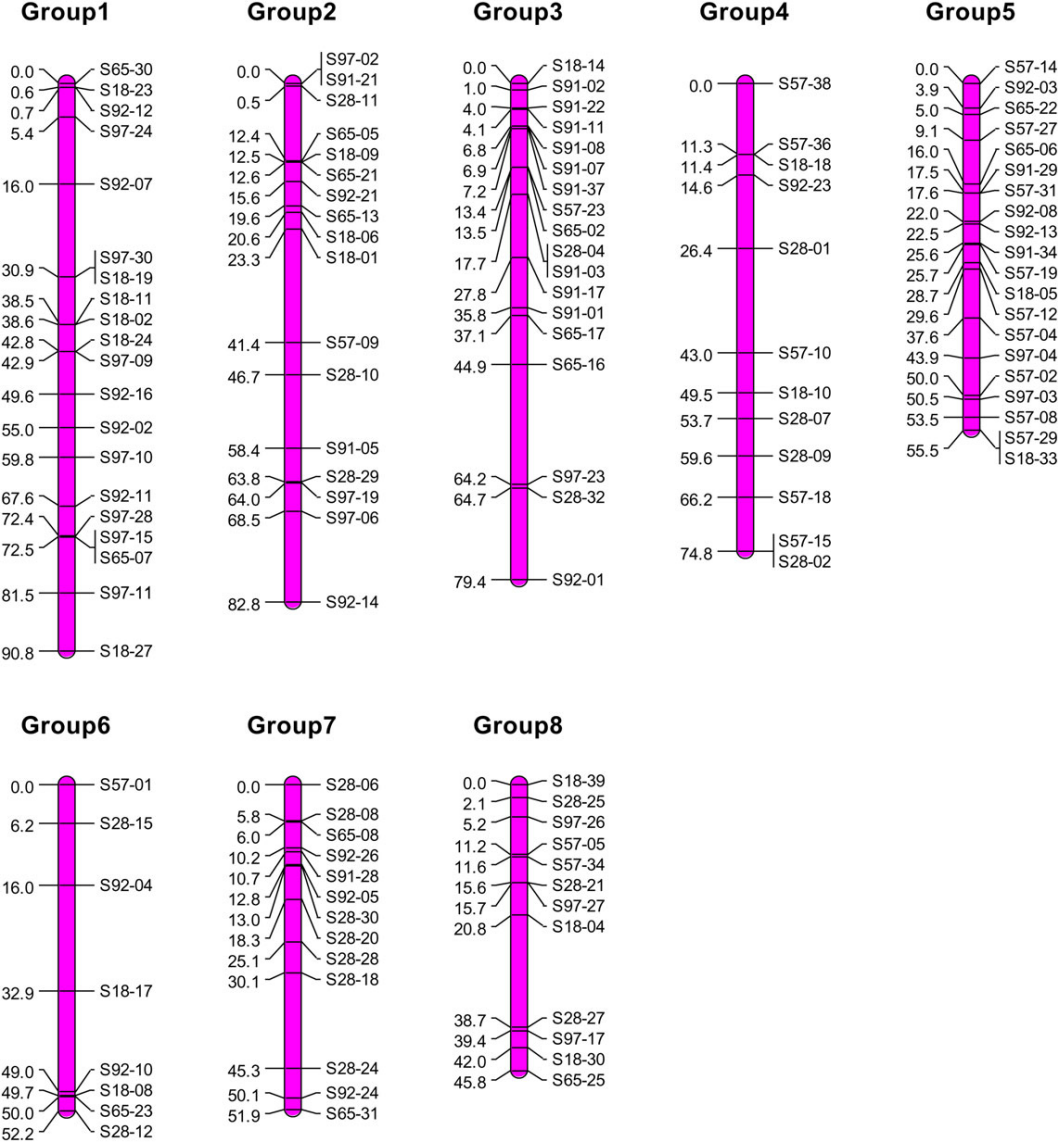
Genetic linkage map consisting of eight linkage groups for the *Torreya* genome constructed by dominant markers. This figure is available in black and white in print and in colour at *DNA Research* online.

Based on the estimated pair-wise recombination fractions, we constructed a genetic linkage map using MapMaker software. Under the thresholds of *θ* = 0.3 and LOD = 3.0, 233 markers were grouped into 8 linkage groups, but with 73 markers unlinked. Markers in each linkage group were ordered with an objective function of the sum of adjacent recombination fractions. When the optimal order of a linkage group was determined, the map distance between any two adjacent markers was calculated by Haldane's map function. To the end, we obtained a low-density genetic linkage map for *T. grandis* (Fig. 1). The total length of the map is 533.2 cM, with an average marker interval of 3.33 cM.

**Figure 2.**
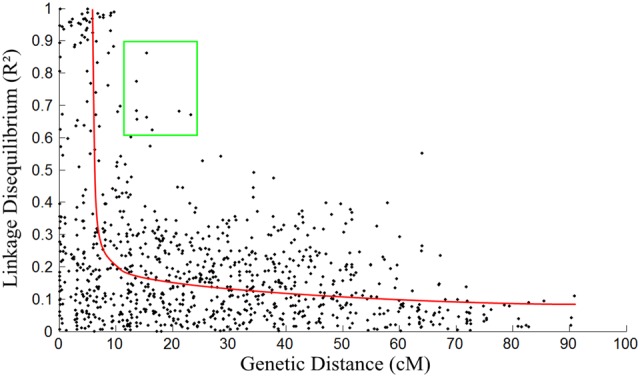
Distribution of normalized linkage disequilibria, expressed as *R*^2^, across genetic distance of the *Torreya* genome in centiMorgans. The curve presents a general trend of the decline of LD with genetic distance. Marker pairs in the square have a large LD, although they are distant by >10 cM from each other. This figure was also used in the study by Sun et al.^23.^ This figure is available in black and white in print and in colour at *DNA Research* online.

By plotting pair-wise LD over the genetic distance, we constructed a LD map from which to infer the population history of *T. grandis* (Fig. 2). In general, the LD declines markedly with marker distance within the first 10 cM of genome, and this decrease quickly becomes gradual after this length. This trend suggests that the population of *T. grandis* sampled may have experienced a long evolutionary history in the environment where this species grows. However, there are quite a few pairs of unlinked markers beyond 10–20 cM of genetic distance which are associated with a large *R*^2^, suggesting that these loci may be subjected to some recent evolutionary forces. Further studies from single-nucleotide polymorphisms (SNPs) are needed to characterize the biological function of these loci and relate this function to possible anthropological selection or climate change towards an in-depth understanding of the evolutionary mechanisms of *T. grandis*.

**Figure 3.**
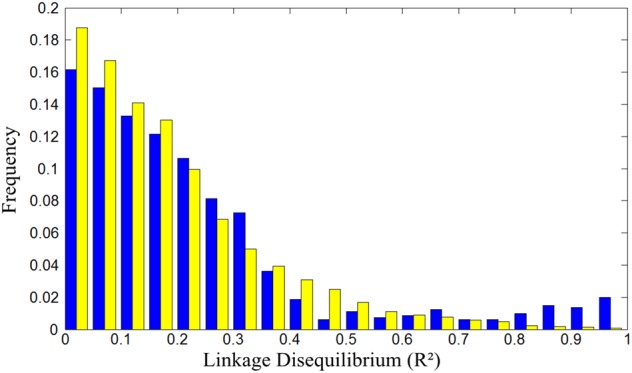
Distribution of LD between markers from the same linkage groups (solid bars) and between markers located on different linkage groups (grey bars) over the *Torreya* genome. This figure is available in black and white in print and in colour at *DNA Research* online.

**Figure 4.**
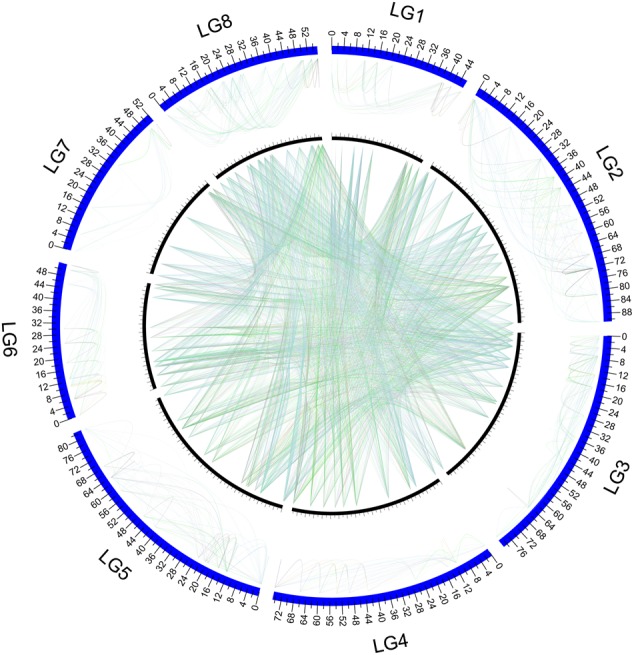
Pattern of LD occurrence between markers from the same linkage groups (outer circle) and from different linkage groups (inner circle). The existence of LD between a pair of markers is denoted by a line that links them, with the magnitude of LD positively related to the thickness of the line. This figure is available in black and white in print and in colour at *DNA Research* online.

From the distribution of all pair-wise LD, it was found that most pairs of markers do not display a large LD value (Fig. 3), conforming to the result inferred from Fig. 2 that this population may have undertaken a long evolutionary history. Although the LD coefficients tend to be larger between markers located within the same linkage group than between markers from different linkage groups (Fig. 3), a portion of between-group markers has a large LD. This suggests that the genome harbouring these markers may be under recent evolutionary forces. Differences in LD occurrence within and among linkage groups are visualized in Fig. 4. It can be seen that markers in linkage Group 7 are not only rarely associated with those from other linkage groups, but also display a sparse distribution of LD with those within the same linkage group. More specifically, of all 13 × 12/2 = 78 possible combinations between 13 markers of this group, only 15 (19.2%) pairs display significant associations. Yet, such percentages for other linkage groups, such as Groups 2 and 8, were observed to be >60%. A reduced frequency of significant associations in linkage Group 7 suggests that this part of the *T. grandis* genome may have experienced a long evolutionary history. Relative to linkage Group 7, linkage Groups 2 and 8 has much more frequent LD between different loci from the same and different linkage groups, implying that some recent pressure of natural selection may have taken place in this part of the genome.

## 4. Computer simulation

To examine the statistical properties of the model for constructing the LD map with dominant markers, we performed computer simulation by mimicking a natural population at HWE. We randomly sample a panel of unrelated open-pollinated families (each including a female parent and multiple offspring). Given a total of 1,000 progeny, the simulation considers three sampling strategies, 1,000 × 1 (1,000 maternals with a single offspring), 200 × 5 (200 maternals with 5 offspring) and 50 × 20 (50 maternals with 20 offspring). For each strategy, we simulated two co-dominant markers with strong and weak LD, *D* = 0.15 and 0.02, respectively, in the population. The allele frequencies for the two markers are *p_A_* = 0.6 vs. *p_a_* = 0.4 and *p_B_* = 0.5 vs. *p_b_* = 0.5, respectively. The two markers are linked with two sizes of then recombination fraction *θ* = 0.20 and 0.05. In each design, 1,000 simulation replicates were performed to estimate the means of the MLEs for each parameter and their standard deviations. By collapsing the simulated co-dominant marker genotypes into a dominant setting, we can test how well our model performs to construct a dominant LD map.

Table 5 gives the results of parameter estimates from simulation studies under different designs. As expected, because of more information contained, co-dominant markers provide better estimation of each parameter than dominant markers, although the drawback of the latter can be overcome by choosing an optimal sampling strategy. General trends of estimation precision of parameters are summarized as follows: (i) LD can be estimated with high accuracy and precision for both co-dominant and dominant markers under all simulation schemes considered. However, as expected, more small families perform better than fewer larger families, because the estimate of LD is based on the sampled parents from the original population. (ii) The estimation of the recombination fraction *θ* is first dependent on the size of LD, followed by the degree of linkage and the sampling strategy. If LD is near zero, then *ϕ* is close to $\frac{1}{2}$ so that *ω*_1_ and *ω*_2_ will not contain *θ*. Thus, *θ* is not estimable when there is no association between the two markers. To better estimate the linkage, precise estimation of LD is essential.

**Table 5.** MLEs (±standard deviations) of allele frequencies, linkage disequilibrium and recombination fraction from 1,000 simulation replicates under different sampling strategies

Family		True	True	MLE			
Number	Size	*D*	*θ*	$\hat{p}$	$\hat{q}$	$\hat{D}$	$\hat{\theta }$
Co-dominant markers							
50	20	0.150	0.200	0.600 ± 0.047	0.502 ± 0.050	0.149 ± 0.023	0.200 ± 0.042
50	20	0.150	0.050	0.600 ± 0.049	0.503 ± 0.050	0.149 ± 0.022	0.051 ± 0.032
50	20	0.020	0.200	0.599 ± 0.049	0.503 ± 0.049	0.020 ± 0.035	0.167 ± 0.155
50	20	0.020	0.050	0.600 ± 0.047	0.503 ± 0.050	0.021 ± 0.036	0.096 ± 0.132
200	5	0.150	0.200	0.599 ± 0.024	0.499 ± 0.025	0.150 ± 0.011	0.200 ± 0.039
200	5	0.150	0.050	0.600 ± 0.024	0.499 ± 0.025	0.150 ± 0.011	0.051 ± 0.030
200	5	0.020	0.200	0.600 ± 0.024	0.500 ± 0.025	0.021 ± 0.018	0.161 ± 0.165
200	5	0.020	0.050	0.600 ± 0.024	0.500 ± 0.025	0.019 ± 0.017	0.107 ± 0.143
1000	1	0.150	0.200	0.600 ± 0.011	0.500 ± 0.011	0.150 ± 0.005	0.200 ± 0.038
1000	1	0.150	0.050	0.600 ± 0.011	0.500 ± 0.011	0.150 ± 0.005	0.049 ± 0.031
1000	1	0.020	0.200	0.600 ± 0.011	0.500 ± 0.012	0.020 ± 0.008	0.172 ± 0.172
1000	1	0.020	0.050	0.600 ± 0.011	0.500 ± 0.012	0.020 ± 0.008	0.113 ± 0.154
Dominant markers							
50	20	0.150	0.200	0.602 ± 0.062	0.505 ± 0.062	0.148 ± 0.035	0.213 ± 0.145
50	20	0.150	0.050	0.604 ± 0.064	0.505 ± 0.061	0.145 ± 0.038	0.093 ± 0.101
50	20	0.020	0.200	0.606 ± 0.066	0.509 ± 0.062	0.009 ± 0.069	0.136 ± 0.166
50	20	0.020	0.050	0.606 ± 0.064	0.506 ± 0.062	0.011 ± 0.068	0.126 ± 0.163
200	5	0.150	0.200	0.601 ± 0.033	0.501 ± 0.031	0.149 ± 0.017	0.200 ± 0.104
200	5	0.150	0.050	0.602 ± 0.033	0.503 ± 0.031	0.149 ± 0.017	0.060 ± 0.060
200	5	0.020	0.200	0.602 ± 0.033	0.502 ± 0.032	0.018 ± 0.028	0.141 ± 0.166
200	5	0.020	0.050	0.602 ± 0.033	0.501 ± 0.031	0.017 ± 0.028	0.135 ± 0.163
1000	1	0.150	0.200	0.600 ± 0.014	0.500 ± 0.013	0.150 ± 0.008	0.202 ± 0.078
1000	1	0.150	0.050	0.600 ± 0.015	0.500 ± 0.014	0.150 ± 0.007	0.055 ± 0.050
1000	1	0.020	0.200	0.600 ± 0.014	0.500 ± 0.014	0.020 ± 0.012	0.132 ± 0.161
1000	1	0.020	0.050	0.600 ± 0.014	0.500 ± 0.014	0.020 ± 0.011	0.118 ± 0.152

An additional scenario of simulation was conducted by collapsing only one of the two co-dominant markers into a dominant status. As expected, this scenario was intermediate in the precision of parameter estimation between those in which both markers are co-dominant and dominant, respectively. We have also performed simulation studies using the same schemes described above, but by quantitatively changing the values of LD within its interval. This simulation allows us to determine the minimum value of LD beyond which *θ* can be well estimated. In the package of software, we provide the function of determining such an LD value given a sampling strategy and allele frequencies.

## 5. Discussion

Similar to the HWE, significant departure from linkage equilibrium (LE) indicates that the population studied is undergoing some evolutionary pressure by extensive inbreeding, gene flow, genetic drift, mutation, natural selection, etc. However, unlike HWE, LE cannot be established in one generation of random mating, rather than it needs a number of generations to be reached, because LD declines at a rate that depends on the recombination fraction.^1^ For this reason, a test of LD about its departure from LE may tell us more stories about the evolutionary history of the population. Just because the use of LD to infer a population's past events is founded on its relationship with the frequency of recombination, a joint estimation of the LD and recombination fraction can provide more precise information about evolutionary inference.^8^

In this article, we extended Wu and Zeng's^8^ open-pollinated progeny design to construct a linkage–LD map and particularly showed how this design can accommodate to missing information of dominant-segregating markers such as cytosine methylation markers. DNA methylation, as a covalent base modification of plant nuclear genomes, is thought to be accurately inherited through both mitotic and meiotic cell divisions.^14^ Also, similarly to spontaneous mutations in DNA, errors in the maintenance of methylation states would violate the equilibrium of natural populations, leading to changes in associations between epialleles at different methylated loci. Thus, by constructing a linkage–LD map using those so-called SMPs, we can infer evolutionary pasts of the natural populations from a different perspective.^23,15^

Indeed, as a simple and cheap technique, dominant markers, such as SRAP markers,^22^ are still being used for many under-represented species including forest trees and wildlife species.^24,25,26^ Thus, the dominant model described can widen the usefulness of the open-pollinated design in practical population studies. Simulation studies have determined an appropriate sampling strategy to construct a linkage–LD map using dominant markers. Since the precise estimation of LD is of primary importance to linkage estimation, we recommend using many smaller families over small larger families. In addition, the efficiency of linkage–LD map construction can be enhanced by three-point analysis, which has proved to not only provide more information about the genome structure and organization, but also reduce a possibility of biased estimation of the linkage when LD has a small value.^27,28^ This is especially true for dominant markers.

Although the original model for joint linkage and LD analysis was proposed more than a decade ago, its practical use has not occurred until recent years when the collection of molecular markers for under-represented species has been feasible. The current study presents one of the first applications of Wu and Zeng's^8^ open-pollinated design to study the population structure and history of an outcrossing species. *Torreya grandis* is a gymnosperm tree species with a large size, endemic to the eastern and southeastern China.^20^ Because of economic and ecological values, this species has been increasingly studied in terms of its evolutionary history and the genetic control of complex traits.^21^ The results from a joint linkage and LD analysis with dominant markers suggest that this species has experienced a long history of evolution, but some regions of its genome are subject to a certain recent evolutionary forces. This information will provide guidance for better germplasm management of this important woody plant. Advances in understanding the evolutionary history of *Torreya* can be made by sampling multiple populations in a range of its distributions. This study, along with the previous one based on half-sib seeds from a single tree of *T. grandis* reporting a linkage map covering a total of 7,139.9 cM in 10 groups,^21^ was among the first to construct genetic linkage maps for genus*.* It is important to align the two maps into a single one for a better coverage of the *Torreya* genome. Also, much more markers that can align those unlinked markers detected from the current and Zeng et al.'s studies are needed to completely cover 11 chromosomes of *T. grandis*. A complete coverage of markers allows more extensive studies of variation and examination of LD patterns, which will better reveal levels of complexity for this species.

It has been recognized that genetic mapping based on LD analysis helps to fine map complex traits or disease, but this approach may have a high likelihood to detect spurious signals of association, because allelic association can also be due to evolutionary forces rather than physical linkage.^2^ A joint linkage and LD analysis can overcome this false-positive discovery.^27^ Thus, the LD map constructed from genetic and epigenetic markers will provide an important fuel to map key QTLs that affect quantitative traits of economic and environmental importance.^7^

## Funding

This work is supported by Special Fund for Forest Scientific Research in the Public Welfare (201404102); Open Fund for Key Discipline of Forestry of Zhejiang Province, Zhejiang A&F University (AF201308); Key Scientific Project of Selection and Breeding of New Agricultural Varieties of the Department of Science and Technologies of Zhejiang Province (2012C12904-12); and ‘One-thousand Person Plan’ Award. Funding to pay the Open Access publication charges for this article was provided by Special Fund for Forest Scientific Research in the Public Welfare (201404102).
